# Supplementary material for: Scalable inference of functional neural connectivity at submillisecond timescales
Source: ArXiv. 2025 Oct 23:arXiv:2510.20966v1. Preprint. [Version 1] (PMC12633633)
Supplement: Supplement 1 [file NIHPP2510.20966v1-supplement-1.pdf]

S Supplement

S.1 Additional details on hippocampal data

S.1.1 Dataset details

The Visual Coding - Neuropixels dataset contains spike time recordings from a variety of regions in the mouse brain, acquired using high-density extracellular electrophysiology probes. During each recording session, the mouse passively views a diverse set of visual stimuli that includes natural images and movies, as well as classical stimuli such as drifting gratings and moving dots [22]. This experimental setup enables simultaneous mapping of visually driven neural activity across different regions of the brain. For our analysis, we selected session 715093703 which includes recorded units from three hippocampal subregions—CA1, CA3, and the dentate gyrus (DG)—to investigate both inter- and intra-regional connectivity.

S.1.2 Analysis details

We restricted our analysis to units recorded with a single probe (probe_id = 810755803), which sampled the three hippocampal subregions (CA1, CA3, DG). Prior to analysis, we also excluded units that emitted fewer than 1000 spikes over the full recording duration (mean firing rate < 0.1 Hz), as connections would be difficult to estimate from such sparse spiking activity. This filtering step reduced the number of units from $N = 117$ to $N = 106$.

We fit the models on three different recording durations: 500 seconds, 1000 seconds, and the full 2.7-hour session. For the 500-second and 1000-second fits, we trained the models on five different subsets of the data and averaged the results across folds. The total number of spikes for each duration was $(2.7 \times 10^5) \pm (8.2 \times 10^3)$, $(5.0 \times 10^5) \pm (5.1 \times 10^4)$, and 4.2×10^6, respectively.

For all model fits, we used the following parameters: ridge regularization strength $\beta = 1000$; 500 training epochs and a sample size of $M = 2 \times 10^6$ for the hybrid PA-MC model, with an adaptive step size determined using backtracking line search. For the discrete PA model, we used a batch size of $B = 300$ seconds. In both polynomial approximation models, the approximation range was set to $[\log(\boldsymbol{\mu}) - 0.3,\ \log(\boldsymbol{\mu}) + 1.2]$, where $\boldsymbol{\mu}$ is the vector of mean firing rates for all neurons.

To determine the mean delay times shown in Table 1 of the main text, we first identified the peak value and corresponding index for each estimated filter. The filters were then normalized by the absolute maximum value across all estimates, ensuring that the largest filter had an amplitude of 1. We then selected filters whose peak index fell within 0.3-2.5 seconds after the presynaptic spike and whose peak amplitude exceeded 0.7.

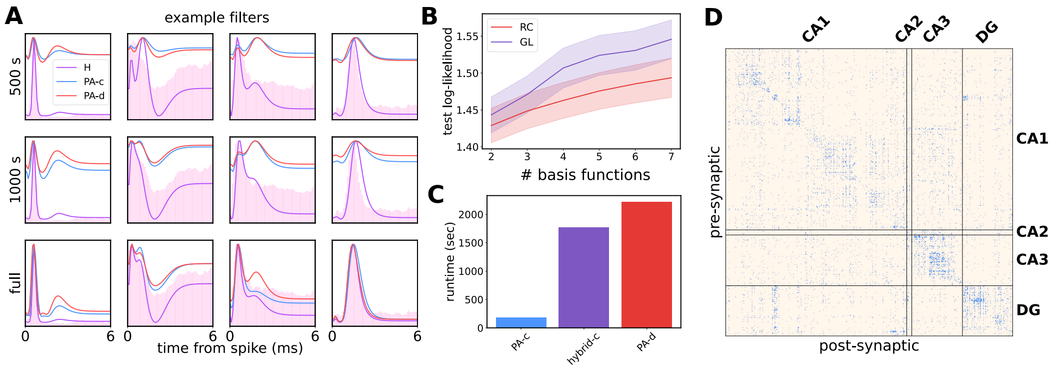

Figure S1: **A** Four example filters estimated from increasingly longer portions of the single-probe hippocampal data; **B** Comparison of the cross-validated log-likelihood on the single-probe dataset using RC and GL bases; **C** Time to completion for polynomial approximation and continuous hybrid models on the multi-probe recording; **D** Putative excitatory connections across all probes.

S.1.3 Additional results

Figure S1**A** shows four example filters estimated at the three data volumes. These examples reflect the MSE values calculated across all filters, as shown in Fig.5**C** of the main text. We show that the PA-based models underperform in low-data regimes but achieve comparable accuracy to the hybrid MC model when trained on the full dataset.

To test the performance of the orthogonal Generalized Laguerre (GL) basis on a complex natural dataset, we perform 5-fold cross-validation on the single probe recording using Generalized Laguerre and raised cosine basis functions with varying numbers of bases ($J = 2, \dots, 7$) and compare the log-likelihood on held-out data. We find that the GL basis consistently outperforms the raised cosine (RC) basis across all tested basis set sizes, with both improving as more functions are added (Fig. S1**B**). The c hyperparameter values used for the different GL basis sizes are $\{0.7, 0.85, 0.95, 1.07, 1.23, 1.36\}$, and $\alpha = 2$ is fixed throughout.

We also provide preliminary results on a 2000-second recording from the full multi-probe dataset ($N = 623$ after excluding low-spiking neurons). In addition to the three hippocampal regions discussed in the main text, this full recording includes a small number of CA2 neurons ($N_{CA2} = 11$), which is insufficient for comprehensive analysis of that subregion's connectivity. Figure S1**C** shows the time to completion for three model fits: PA-c is by far the fastest, followed by the hybrid PA-MC model and the discrete polynomial approximation (PA-d). The connectivity matrix inferred from the full population exhibits a block structure that aligns with probe boundaries: neurons on the same probe are more likely to have identified connections than across probes (Fig. S1**D**). These artifacts—likely arising from systematic differences in recording quality, spatial sampling, or local network properties across probes—motivated our decision to focus the main text analysis on a single-probe recording.

S.2 Additional details on simulated data

S.2.1 Simulation

In this work, we used data simulated from both one-to-all and all-to-all coupled GLMs. We employed a two-step procedure to generate spike trains from an inhomogeneous Poisson point process. First, we simulated discrete-time binned spike counts for the postsynaptic neuron(s) using a Poisson GLM:

$$\begin{aligned} \boldsymbol{\lambda}_t &= \exp(\mathbf{w}^\top \boldsymbol{x}_t + \mathbf{b}) \\ \mathbf{y}_t &\sim \text{Poisson}(\boldsymbol{\lambda}_t) \end{aligned}$$

Here, $\boldsymbol{x}_t$ is a row of the design matrix representing presynaptic spike counts at time t, and $\mathbf{b}$ is a vector of background log-rates per bin, i.e $\mathbf{b} = \log(\lambda_b \cdot \delta t)$ where δt is the bin size. The simulation bin size was set to 0.05 ms, which is smaller than the 0.1 ms bin size used during inference. This choice minimizes discretization artifacts, ensuring there is at most one spike per bin, and avoids introducing bias that could favor discrete-time models whose structure would otherwise align exactly with the simulation binning.

Next, we converted the binned spike counts into continuous spike times by sampling them uniformly within each bin, leveraging the memoryless property of interarrival times in Poisson point process:

$$s_t \sim \text{Uniform}(t, t + \delta t)$$

where s_t is the spike time in bin t. For discrete-time model fitting, we re-binned the resulting continuous spike times at the inference resolution (0.1 ms), effectively introducing slight temporal jitter.

S.2.2 All-to-one simulation

We simulated data using 100 raised cosine (RC) basis functions with a cosine bump width set to 25 and a log-scaling constant of 300. Synaptic weights were drawn independently from a Gaussian distribution with zero mean and standard deviation $\sigma_w = 0.4$ setting the overall strength of the couplings. Presynaptic neurons fired with constant rates sampled from a normal distribution with mean $\mu = 10$ Hz and standard deviation $\sigma_\lambda = 1.0$. These presynaptic spike times were used to

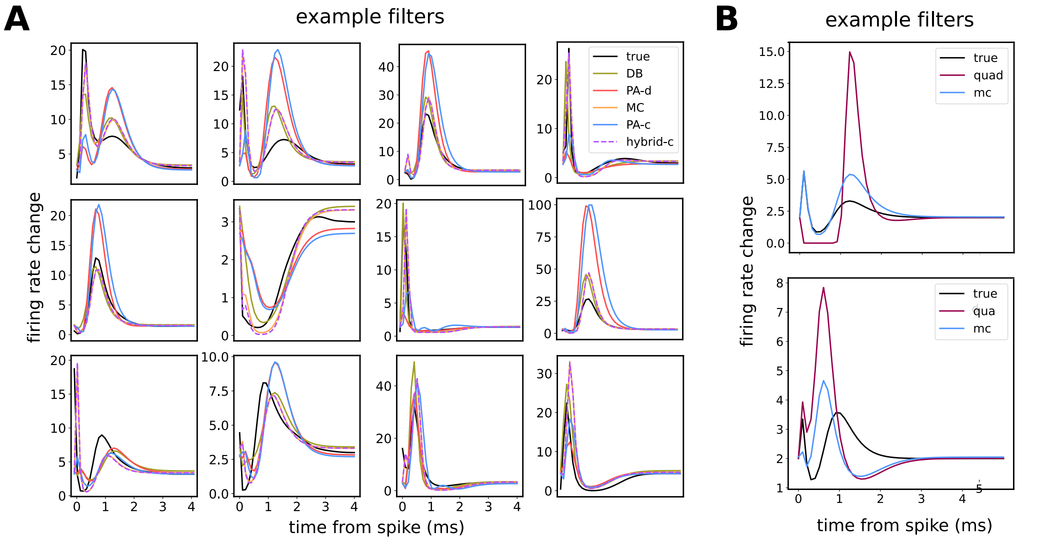

Figure S2: **A** Example filters inferred by all models trained on simulated data; **B** Example filters estimated using MC sampling and Gauss-Lobatto quadrature nodes.

model the activity of a single postsynaptic neuron. No post-spike (self-history) filter was included in this setup. For these simulations, we varied the recording length $T \in \{10, 40, 160, 650, 2500, 10^4\}$ seconds, approximately following exponential growth. The number of presynaptic neurons was fixed at $N = 8$. For each recording length, we simulated 15 datasets with different random weights and averaged time to completion and MSE across model fits.

All models were fit using a generalized Laguerre polynomial basis with $J = 4$, $c = 1.5$ and $\alpha = 1$. The MC model was trained for 3000 epochs with 5×10^5 MC samples per update, while the hybrid PA-MC model was trained for 1000 epochs using 10^6 samples. The DB model was trained for $E_T \in \{1000, 1000, 400, 200, 100, 50\}$ epochs corresponding to the recording lengths listed above. For both discrete-time models (PA-d and DB), the number of batches was chosen between 2 and 25, with approximate batch size $B = 4 \times 10^6$ time bins. Figure S2**A** shows example filters for all five models fit on 10^4 seconds of the recording and selected from multiple simulation runs. The inferred filters generally capture the amplitudes and temporal structure of the generative filters, though they do not match them exactly. This discrepancy is expected due to the distributional mismatch between simulation and inference.

S.2.3 All-to-all simulation

All-to-all simulations used the same set of 100 RC basis functions. Synaptic connectivity was sparse, with a connection probability set to $p = 0.1$, and coupling weights drawn from a zero-mean Gaussian distribution with $\sigma_w = 0.2$. Excitatory and inhibitory connections were randomly assigned with a ratio of 80% to 20%, respectively. Baseline firing rates for all neurons were sampled from a normal distribution: $\lambda_b \sim \mathcal{N}(3, 0.5)$. These simulations ran for a fixed duration of 100 seconds, while varying the population size: $N \in \{10, 35, 100, 350\}$. We used the same GL basis to fit all models on this dataset, without applying additional regularization. The MC and DB models were trained for $E_N \in \{3000, 3000, 2000, 600\}$ epochs, corresponding to each population size listed above, while hybrid PA-MC model was trained for half as many epochs. The number of training batches for the discrete models was chosen between 2 and 50 depending on the population size.

S.2.4 Stochastic optimization

Throughout this work, unless stated otherwise, all analyses use JAXopt's gradient descent solver with default settings and perform gradient updates explicitly in a loop. For the continuous-time gradient-optimized models (MC and hybrid PA-MC), we determine convergence based on the gradient step norm $u_t = \|\eta_t \cdot \nabla \mathcal{L}(\theta_t)\|$, which is available directly from the optimizer state and therefore computing it requires no additional cost. We decide the model has converged when u_t does not

decrease for 100 consequent steps. For the discrete batched (DB) model, u_t computed on batched updates is meaningless, so instead we evaluate the training log-likelihood on the full dataset at the end of each epoch. In Fig. 3**A,B** of the main text, we show negative log-likelihood (objective) function values for the DB and MC models. Note that these values are on different scales: the discrete model reports mean negative log-likelihood per time bin, while the continuous approach uses the integrated (summed) negative log-likelihood over the entire recording. More generally, negative log-likelihood values for discrete and continuous distributions are not directly comparable.

S.3 Comparison to quadrature approach

As discussed in the main text, [17] is the only prior work that fits a continuous-time Poisson GLM, using Gauss-Lobatto quadrature to approximate the conditional intensity function (CIF) integral in the log-likelihood. Their approach requires inserting a varying number of quadrature nodes between every pair of consecutive spike times. For our hippocampal dataset with approximately 5 million spikes, this would necessitate storing and evaluating far more nodes than spikes, leading to severe memory constraints and prohibitively slow inference. In contrast, our Monte Carlo (MC) approach uses many times fewer samples than the number of spikes in the dataset, making it substantially more memory-efficient and faster. Unfortunately, the code repository referenced in [17] is no longer publicly available, which limited our ability to leverage potential optimizations in their original implementation.

To assess the quadrature approach under more favorable conditions, we implemented it on a small simulated dataset ($N = 8$ neurons, $T = 100$ seconds) where computational constraints are not prohibitive. Following the approach in [17], we set the total number of nodes for quadrature integration to 50 nodes per second of recording, with a minimum of 3 nodes per inter-spike interval and the rest distributed proportionally to interval length. Figure S2**B** shows example filter fits from the quadrature and MC methods on this simulated dataset run for only 100 gradient steps. Across all filters, the MC approach achieved substantially lower mean squared error (MSE $= 2.54 \pm 0.75$) compared to the quadrature approach (MSE $= 5.37 \pm 0.48$). We believe this reduced accuracy is due to the high-frequency content of the coupling filters, which cannot be captured well by standard low-order quadrature schemes that assume smooth integrands. Notably, the quadrature method required approximately 2.5 hours to fit, compared to $\sim$20 seconds for the MC approach—a more than 400-fold difference in runtime.

These results highlight practical barriers to applying existing quadrature-based methods to large-scale neural datasets. Developing more scalable quadrature methods—those better suited for GPU acceleration or for estimating high-frequency coupling filters—remains a promising direction for future work.

S.4 Softplus nonlinearity

Both continuous-time approaches introduced in this paper—Monte Carlo (MC) and polynomial approximation (PA)—can be extended to use inverse link functions Φ beyond the standard exponential nonlinearity. A commonly used alternative in Poisson GLM is the softplus function, $\text{softplus}(x) = \log(1 + \exp(x))$, which provides improved numerical stability due to its bounded gradient and slower growth at large inputs. In the MC approach, incorporating a different inverse link function is straightforward: the model simply uses the new Φ when computing both the first log-likelihood term and the MC estimate of the CIF. In the PA approach, an additional motivation for using softplus is that it can be more accurately approximated by a second-order polynomial than the exponential function, especially over a wider range of inputs, as pointed out in [4, 19]. Figure S3**A** shows second-order Chebyshev approximations to firing rate range $1 - 5$ Hz (top row) and $0.5 - 10$ Hz (bottom row). However, using softplus introduces an additional nonlinearity into the first term of the log-likelihood. While prior work [4] addresses this by introducing a second Chebyshev approximation for $\log(\text{softplus}(\cdot))$, we propose an alternative that avoids this extra approximation step at the cost of increased computation time. Specifically, we continue to approximate the CIF with a quadratic, but evaluate the first log-likelihood term exactly using the intensity function $\lambda(t)$:

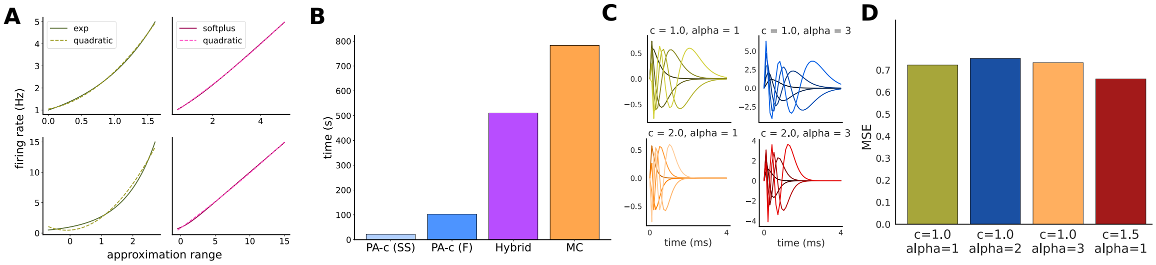

Figure S3: **A** Polynomial approximations to exp and softplus nonlinearities across different firing rate ranges; **B** Runtime comparison for the continuous models using softplus inverse link; **C** Visualization of the GL basis for varying hyperparameter choices; **D** Mean squared error (MSE) relative to true simulated filters for bases sets in **C**.

$$\begin{aligned}\log p(\mathbf{y} \mid \mathbf{X}, \boldsymbol{w}) &= \sum_{k=1}^{K} \log \lambda(y_k) - \int_0^T \lambda(t)dt \\ &\approx \left[\sum_{k=1}^{K} \log\, \Phi\left(\sum_{t_s \in \mathcal{X}_n(y_k, H)} \boldsymbol{w}_n^\top \boldsymbol{\phi}(y_k - t_s)\right)\right] - a_2 \boldsymbol{w}^\top \mathbf{M} \boldsymbol{w} - a_1 \boldsymbol{w}^\top \mathbf{m}\end{aligned} \tag{12}$$

This modified objective allows standard gradient-based optimization. Since the likelihood is deterministic and only the first term must be recomputed at each gradient step, convergence to the PA closed-form solution remains significantly faster than for the MC approach, despite the added cost of computing the softplus exactly in the first term. In Figure S2**B**, we compare the runtime of computing PA-c sufficient statistics (which approximates the time required to obtain a closed-form solution), fitting the full PA-c model using an L-BFGS solver, training the hybrid PA-MC model, and training an MC model from scratch. All fits were performed on the full hippocampal dataset used in this paper.

S.5 Additional details on generalized Laguerre polynomials

We define a set of J basis functions using generalized Laguerre polynomials of increasing order: $\phi = (L_0^{(\alpha)}, L_1^{(\alpha)}, \ldots, L_J^{(\alpha)})$. All functions are evaluated on an empirically selected interval $[0, 30]$, which can be mapped to any history window $[0, W]$. The rise and decay times, as well as the amplitude of the basis functions, are controlled by manipulating hyperparameters c and α (Fig.S3**C**). For our analyses, we fix $c = 1.5$, which ensures that for $J \in [4, 5]$ the last basis function decays to zero near the end of the history window. This is similar to how the raised cosine basis achieves this by shifting each cosine bump relative to the start of the support. While c can be adjusted for other values of J, we find that the models are robust to changes in c and α and varying them has little effect on the resulting MSE values (Fig.S3**D**).

In continuous-time PA, we must compute integrals of individual basis functions, φ_j, and integrals of pairwise products over interaction ranges, $\left[M_{t_s, t_{s'}}\right]_{j,j'}$. GL bases offers an advantage of computing them efficiently in a closed-form. While RC bases also admit analytical solutions in principle, we find that determining the correct integration bounds is non-trivial because each cosine bump has limited support $[d_i - \pi, d_i + \pi]$ where d_i is the center of the bump. In contrast, GL basis functions are defined over the full history window $[0, W]$.

The integral of a single GL basis function is:

$$
\begin{aligned}
\varphi_j &= \int_0^W \phi_j(\tau)\, d\tau = \int_0^W L_j^{(\alpha)}(c\tau)\, e^{-c\tau/2}(c\tau)^{\alpha/2}\, d\tau \\
&= \sum_{k=0}^{j} \binom{j+\alpha}{j-k} \frac{(-1)^k}{k!} \int_0^W (c\tau)^{k+\frac{\alpha}{2}} e^{-c\tau/2}\, d\tau \\
&= \sum_{k=0}^{j} C_k \int_0^{\frac{cW}{2}} \frac{c^{k+\frac{\alpha}{2}} \cdot 2^{k+\frac{\alpha}{2}+1}}{c^{k+\frac{\alpha}{2}+1}} u^{k+\frac{\alpha}{2}} e^{-u}\, du \quad (\text{where } u = \tfrac{c\tau}{2}) \\
&= \sum_{k=0}^{j} C_k \cdot \frac{2^{k+\frac{\alpha}{2}+1}}{c}\, \gamma\big(k + \tfrac{\alpha}{2} + 1, \tfrac{cW}{2}\big)
\end{aligned}
\tag{13}
$$

where $C_k = \binom{j+\alpha}{j-k}\frac{(-1)^k}{k!}$ and $\gamma(a,x) = \int_0^x t^{a-1}e^{-t}\, dt$ is the lower incomplete gamma function.

The pairwise interaction integral is given by:

$$
\begin{aligned}
\big[M_{t_s,t_{s'}}\big]_{j,j'} &= \int_\delta^W \phi_j(\tau)\phi_{j'}(\tau-\delta)\, d\tau \\
&= \int_\delta^W L_j^{(\alpha)}(c\tau)e^{-c\tau/2}(c\tau)^{\alpha/2} \cdot L_{j'}^{(\alpha)}(c(\tau-\delta))\, e^{-c(\tau-\delta)/2}(c(\tau-\delta))^{\alpha/2}\, d\tau \\
&= e^{c\delta/2} \sum_{k=0}^{j}\sum_{k'=0}^{j'} C_k C_{k'} \int_0^{W-\delta} (c(u+\delta))^{k+\frac{\alpha}{2}} (cu)^{k'+\frac{\alpha}{2}} e^{-cu}\, du \quad (\text{where } u = \tau - \delta) \\
&= e^{-c\delta/2} \sum_{k=0}^{j}\sum_{k'=0}^{j'} C_k C_{k'} \sum_{r=0}^{[k+\alpha/2]} \binom{k+\alpha/2}{r} \delta^{k+\frac{\alpha}{2}-r} \int_0^{W-\delta} c^{k+k'+\alpha} u^{r+k'+\frac{\alpha}{2}} e^{-cu}\, du \\
&= e^{-c\delta/2} \sum_{k=0}^{j}\sum_{k'=0}^{j'} C_k C_{k'} \sum_{r=0}^{[k+\alpha/2]} C_r \int_0^{c(W-\delta)} \frac{c^{k+k'+\alpha}}{c^{r+k'+\frac{\alpha}{2}+1}} v^{r+k'+\frac{\alpha}{2}} e^{-v}\, dv \quad (\text{where } v = cu) \\
&= e^{-c\delta/2} \sum_{k=0}^{j}\sum_{k'=0}^{j'} C_k C_{k'} \sum_{r=0}^{[k+\alpha/2]} C_r\, c^{k+\frac{\alpha}{2}-r-1}\, \gamma\big(r + k' + \tfrac{\alpha}{2} + 1,\, c(W-\delta)\big)
\end{aligned}
\tag{14}
$$

where $C_{k'} = \binom{j'+\alpha}{j'-k'}\frac{(-1)^{k'}}{k'!}$, $C_r = \binom{k+\alpha}{r}\delta^{k+\frac{\alpha}{2}-r}$ and $\delta = \delta_{t_{s,n},t_{s',n'}}$ is the spike time difference.

S.6 Polynomial Approximation to Poisson point process log-likelihood

S.6.1 Full derivation

We derive a polynomial approximation to the continuous-time GLM by approximating the nonlinearity Φ with a quadratic function. This allows us to express the cumulative intensity function (CIF) as a sum of precomputable terms:

$$
\begin{aligned}
\int_0^T \lambda(t) = \int_0^T \Phi\left(\sum_n \sum_{t_s\in\mathcal{X}_n} \boldsymbol{w}_n^\top \boldsymbol{\phi}(t-t_s)\right) dt \approx \int_0^T a_2 \left(\sum_n \sum_{t_s\in\mathcal{X}_n} \boldsymbol{w}_n^\top \boldsymbol{\phi}(t-t_s)\right)^2 dt \\
+ \int_0^T a_1 \sum_n \sum_{t_s\in\mathcal{X}_n} \boldsymbol{w}_n^\top \boldsymbol{\phi}(t-t_s)\, dt \\
+ T a_0
\end{aligned}
\tag{15}
$$

where $\mathbf{w}_n \in \mathbb{R}^J$ is the subvector of model parameters corresponding to presynaptic neuron n, and S_n is the set of its spike times. The coefficients a_0, a_1, a_2 parametrize a Chebyshev polynoimal that approximates the true nonlinearity Φ by minimizing MSE over a specified range.

Linear term

Since the basis functions $\boldsymbol{\phi} : [0, W] \mapsto \mathbb{R}^J$ have compact time support in the history window $[0, W]$, we can simplify the linear term:

$$\begin{aligned}\int_0^T a_1 \sum_n \sum_{t_s \in \mathcal{X}_n} \sum_j w_{nj} \phi_j(t - t_s)\, dt &= a_1 \sum_n \sum_{t_s \in \mathcal{X}_n} \sum_j w_{nj} \int_{t_s}^{t_s + H} \phi_j(t - t_s)\, dt \\ &= a_1 \sum_n \sum_{t_s \in \mathcal{X}_n} \boldsymbol{w}_n^\top \boldsymbol{\varphi}\end{aligned} \tag{16}$$

where $\boldsymbol{\varphi} \in \mathbb{R}^J$ with entries $\varphi_j = \int_0^W \phi(\tau)\, d\tau$ (we substitute $\tau = t - t_s$) concatenates the integrals of all basis functions. Due to linearity, the relative timing of the spikes does not matter, only their total number, meaning we can define the linear sufficient statistic vector:

$$\mathbf{m} = \begin{bmatrix} S_1 \boldsymbol{\varphi} \\ S_2 \boldsymbol{\varphi} \\ \vdots \\ S_N \boldsymbol{\varphi} \end{bmatrix} \in \mathbb{R}^{NJ} \tag{17}$$

where S_n is the number of spikes from neuron n, and rewrite the linear term compactly as:

$$a_1 \mathbf{m}^\top \mathbf{w}$$

Quadratic term

Next, we address the second-order term by expanding the squared sum inside the integral::

$$\begin{aligned}\int_0^T a_2 \left(\sum_n \sum_{t_s \in \mathcal{X}_n} \boldsymbol{w}_n^\top \boldsymbol{\phi}(t - t_s) \right)^2 dt &= a_2 \sum_{n,n'} \boldsymbol{w}_n^\top \left(\int_0^T \sum_{\substack{t_s \in \mathcal{X}_n \\ t_{s'} \in \mathcal{X}_{n'}}} \boldsymbol{\phi}(t - t_s) \boldsymbol{\phi}(t - t_{s'})^\top dt \right) \boldsymbol{w}_{n'} \\ &= a_2 \sum_{n,n'} \boldsymbol{w}_n^\top \mathbf{M}_{n,n'} \boldsymbol{w}_{n'},\end{aligned} \tag{18}$$

where we define the neuron-pair interaction matrices:

$$\mathbf{M}_{n,n'} = \sum_{\substack{t_s \in \mathcal{X}_n \\ t_{s'} \in \mathcal{X}_{n'}}} \mathbf{M}_{t_s, t_{s'}}, \quad \text{with} \quad \mathbf{M}_{t_s, t_{s'}} = \int_0^T \boldsymbol{\phi}(t - t_s) \boldsymbol{\phi}(t - t_{s'})^\top dt.$$

For each pair of spikes, we compute the difference $\delta_{t_s, t_{s'}} = |t_{s'} - t_s|$ and note that the integral is nonzero only when $\delta_{t_s, t_{s'}} \leq W$, i.e. when the spikes' contributions overlap within the history window. Making the substitution $\tau = t - t_s$ again, we obtain the entries:

$$\left[\mathbf{M}_{t_s, t_{s'}}\right]_{j,j'} = \int_{\delta_{t_s, t_{s'}}}^{H} \phi_j(\tau) \phi_{j'}(\tau - \delta_{t_s, t_{s'}})\, d\tau \tag{19}$$

The full interaction matrix $\mathbf{M} \in \mathbb{R}^{NJ \times NJ}$ is block-structured with the (n, n')-th block given by $\mathbf{M}_{n,n'}$. This matrix is symmetric because for each spike pair, the interaction matrix satisfies $\mathbf{M}_{t_s, t_{s'}} = \mathbf{M}_{t_{s'}, t_s}^\top$ (the transpose is due to the substitution $\tau = t - t_s$), which implies $\mathbf{M}_{n,n'} = \mathbf{M}_{n',n}^\top$. Thus, the quadratic term becomes:

$$a_2 \boldsymbol{w}^\top \mathbf{M} \boldsymbol{w}.$$

Combining the linear and quadratic terms, the CIF is now approximated with a quadratic function of the weights:

$$\int_0^T \Phi \left(\sum_n \sum_{t_s \in \mathcal{X}_n} \boldsymbol{w}_n^\top \boldsymbol{\phi}(t - t_s) \right) dt \approx a_2 \boldsymbol{w}^\top \mathbf{M} \boldsymbol{w} + a_1 \mathbf{m}^\top \boldsymbol{w} + T a_0 \tag{20}$$

Full log-likelihood approximation

For the Poisson point process log-likelihood, the first term $\sum_{k=1}^{K} \log(\lambda(y_k))$ remains linear in $\mathbf{w}$ when $\Phi = \exp$. As with $\mathbf{m}$, we precompute a vector $\mathbf{k} \in \mathbb{R}^{NJ}$, aggregating the presynaptic contributions at each postsynaptic spike time y_k:

$$\begin{aligned} \sum_{k=1}^{K} \sum_n \sum_{\substack{t_s \in \\ \mathcal{X}_n(y_k, H)}} \boldsymbol{w}_n^\top \boldsymbol{\phi}(y_k - t_s) &= \sum_n \boldsymbol{w}_n^\top \left(\sum_{k=1}^{K} \sum_{\substack{t_s \in \\ \mathcal{X}_n(y_k, H)}} \boldsymbol{\phi}(y_k - t_s) \right) \\ &= \sum_n \boldsymbol{w}_n^\top \boldsymbol{\psi}_n = \boldsymbol{w}^\top \mathbf{k} \end{aligned} \tag{21}$$

where $\mathcal{X}_n(y_k, H)$ contains spikes from neuron n falling within the history window before y_k and $\mathbf{k} = [\boldsymbol{\psi}_1^\top, \boldsymbol{\psi}_2^\top, \ldots, \boldsymbol{\psi}_N^\top]$.

Putting it all together, we approximte the log-likelihood is:

$$\begin{aligned} \log p(\mathbf{y} \mid \mathbf{X}, \boldsymbol{w}) &= \sum_{k=1}^{K} \log \lambda(y_k) - \int_0^T \lambda(t) dt \\ &\approx \boldsymbol{w}^\top \left(\mathbf{k} - a_1 \mathbf{m} \right) - a_2 \boldsymbol{w}^\top \mathbf{M} \boldsymbol{w} \end{aligned} \tag{22}$$

This quadratic formulation admits a straightforward closed-from solution to maximum likelihood estimate (MLE) of the parameters:

$$\tilde{\boldsymbol{w}}_{MLE} = (2 a_2 \mathbf{M})^{-1} (\mathbf{k} - a_1 \mathbf{m}). \tag{23}$$

S.6.2 Comparison of discrete and continuous sufficient statistics

In the discrete polynomial approximation approach described in [4], the linear and quadratic sufficient statistics are given by $\sum_{t=1}^{T} \boldsymbol{x}_t$ and $\sum_{t=1}^{T} \boldsymbol{x}_t \boldsymbol{x}_t^\top$, respectively. Here, $\mathbf{X} \in \mathbb{R}^{T \times NJ}$ is the design matrix formed by convolving binned spike counts with basis function kernels. Each row $\boldsymbol{x}_t \in \mathbb{R}^{NJ}$ encodes the sum of basis function evaluations at time t for all presynaptic spikes $t_s \in \mathcal{X}_n$ such that $t - t_s \leq H$, i.e. those that fall within the basis functions' support window. These quantities can therefore be directly related to the continuous-time approximation sufficient statistics $\mathbf{m}$ and $\mathbf{M}$, up to a scaling factor of the bin size.

The accuracy of this discretization depends on the temporal resolution of the binning: coarser time bins introduce additional approximation error in the computation of the sufficient statistics. This effect is particularly pronounced for basis functions with sharp temporal features or high-frequency components, which includes both log-scaled raised cosine bases and generalized Laguerre polynomials.

S.7 Compute resources

All simulation runs were performed using 16 Intel Ice Lake CPU cores. Analyses on simulated data were conducted using a single NVIDIA A100 GPU with 40 GB of memory. Analyses on real data used a single NVIDIA A100 GPU with 80 GB of memory to increase parallelization capacity.
